# Seroprevalence and associated factors of* Leishmania infantum* in small ruminants in Portugal

**DOI:** 10.1186/s13071-026-07424-y

**Published:** 2026-05-06

**Authors:** Hélder Quintas, María Eugenia Lebrero, João Jacob-Ferreira, Pablo Quilez, David Guallar, Ana Cláudia Coelho, Delia Lacasta, Diana Marteles-Aragüés, Héctor Ruíz, Marta Ruiz de Arcaute, Luís Cardoso, Sergio Villanueva-Saz

**Affiliations:** 1 Mountain Research Centre (CIMO), School of Agriculture, Polytechnic Institute of Bragança (IPB), Campus de Santa Apolónia, 5300-253 Bragança, Portugal; 2 Animal Pathology Department, Veterinary Faculty and Instituto Agroalimentario de Aragón—IA2, Universidad de Zaragoza–CITA, Miguel Servet 177, 50013 Saragossa, Spain; 3 Clinical Immunology Laboratory, Veterinary Faculty, University of Zaragoza, 50013 Saragossa, Spain; 4 Ruminant Clinical Service, Veterinary Faculty, University of Zaragoza, Miguel Servet 177, 50013 Saragossa, Spain; 5 Department of Veterinary Sciences, and Animal and Veterinary Research Centre (CECAV), University of Trás-os-Montes e Alto Douro (UTAD), 5000-801 Vila Real, Portugal; 6 Associate Laboratory for Animal and Veterinary Sciences (AL4AnimalS), Vila Real, Portugal

**Keywords:** Goat, ELISA, *Leishmania infantum*, Portugal, Serology, Sheep

## Abstract

**Background:**

Leishmaniosis is a vector-borne disease caused by *Leishmania* parasites transmitted by infected phlebotomine sand flies. In the epidemiological study reported here, we investigated exposure to *Leishmania infantum* in sheep (*Ovis aries*) and goats (*Capra hircus*) in mainland Portugal.

**Methods:**

A cross-sectional serosurvey was conducted in three geographical regions of Portugal (Trás-os-Montes, Centre and South), with sampling in 19 municipalities and 87 localities.

**Results:**

A total of 2124 small ruminants were tested (1820 sheep and 304 goats). Overall seroprevalence was 21.3% (453/2124; 95% confidence interval [CI] 19.6–23.1), with higher seropositivity in sheep than goats (22.4% vs 15.1%). Univariable analysis showed that ruminant species and reported contact between goats and sheep were statistically associated with seropositivity, but these associations were not detected in the subset of 1851 animals used for multivariable modelling. Rather, multivariable logistic regression analysis (*n* = 1851) showed that several management and biosecurity factors were linked to an increased risk of infection. Increased odds of infection were observed in non-autochthonous breeds (odds ratio [OR] 2.163), holdings without goat–sheep contact (OR 1.920), high aeration/ventilation (OR 1.964), suboptimal drinking fountain hygiene (OR up to 6.221), biannual versus annual disinfection (OR 2.459) and not using equipment from other farms (OR 2.189). Permanent confinement was protective (OR 0.415).

**Conclusions:**

These results indicate widespread exposure of Portuguese flocks to *L. infantum,* highlighting the relevance of husbandry practices. Further research is needed to determine whether sheep and goats develop clinical disease and to clarify the implications for animal and public health.

**Graphical Abstract:**

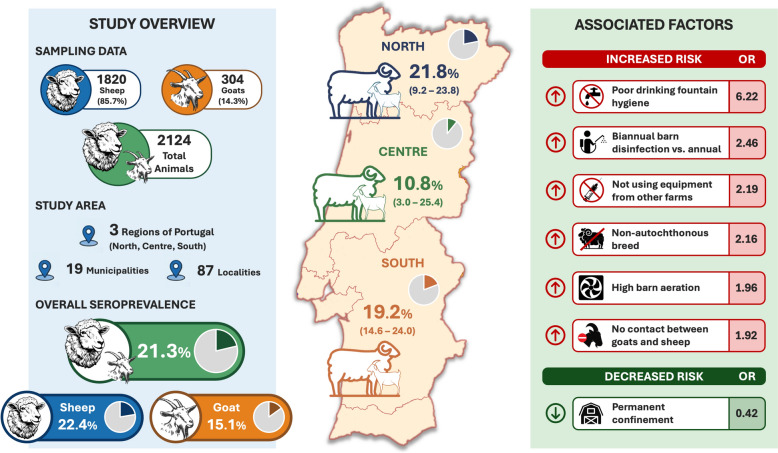

## Background

*Leishmania* is a genus of protozoan parasites belonging to the family Trypanosomatidae. These parasites are transmitted mostly through the bite of infected female phlebotomine sand flies, which feed on blood to produce eggs, causing a disease known as leishmaniosis in 70 vertebrate host species, including humans [1]. Leishmaniosis is considered to be an endemic disease in 99 countries worldwide, including southern European countries such as Greece, Italy, France, Spain and Portugal [1].

Although there are different species of *Leishmania*, *Leishmania infantum* is autochthonous in every country within the Mediterranean biogeographical region and the Balkan countries, with highly variable occurrence [2]. Canids, particularly domestic dogs, are considered to be the main reservoir for *L. infantum* in the Mediterranean Basin and play a crucial role in the domestic cycle of the parasite [3]. Leishmaniosis is also a zoonotic disease with three main clinical forms in humans: visceral (the most severe, as it is almost always fatal without treatment), cutaneous (the most common, generally causing skin ulcers) and mucocutaneous (affecting the mouth, nose and throat) [2]. Since it is a disease affecting multiple animal species, areas with a high prevalence of infection in animals or high prevalence of vectors are directly related to a high incidence of leishmaniosis in humans [4].

It is recognised that several domestic species, such as goat [5] and horse [6], as well as wild mammals, including endangered species such as the European mink [7] and various zoo animals [8–10], and humans, can be considered potential hosts and have been recorded to be naturally infected with *L. infantum* [11]. In recent years, some studies have demonstrated an increase in both infection prevalence and seroprevalence in cats [12] and the presence of infection in ferrets [13, 14] and wild rabbits [15], among others. Nevertheless, the true role of these animals, as opposed to that of dogs, is not well described and remains under discussion in the endemic areas.

Currently, scientific literature on the role of livestock, such as horses or domestic ruminants, in the maintenance and spread of *L. infantum* in European countries is scarce and only a limited number of reports have been published [16]. Recent studies have detected antibodies against *L. infantum* in sheep in Spain, an endemic area, ranging from 9.27% [17] to 15.4% [18] of sheep tested, and also in southern Germany, a non-endemic area, where around 2% of the tested sheep presented antibodies [19]. The presence of antibodies against *L. infantum* in these small ruminants, along with the first European report from Spain of a goat showing clinical signs of leishmaniosis due to *L. infantum* infection is noteworthy. The goat tested exhibited skin lesions for several months and tested positive for antibodies against *L. infantum*; after a common treatment for canine leishmaniosis, the goat made a full recovery [5], raising the question of the possible role of sheep in the parasite's life-cycle and their potential as reservoirs of the infection. Related to the seroprevalence level in goats in Spain, a recent study detected a level of 7.3% in Granada Province located in the southeast of the Iberian Peninsula [18].

In Portugal, *L. infantum* infection is endemic and canine leishmaniosis remains a relevant veterinary and public health concern, with marked geographical heterogeneity. A recent nationwide cross-sectional survey of client-owned dogs sampled in 2021 reported a true seroprevalence of approximately 12.5% [20]. In humans, visceral leishmaniasis is considered to be hypoendemic but persists, with several cases reported annually through mandatory notification [21]. This epidemiological scenario, consisting of spatially heterogeneous canine exposure and continued human disease, supports the need to better characterise potential additional hosts that might contribute to parasite circulation locally, particularly in rural environments where vectors, dogs, livestock and humans coexist.

Given the absence of epidemiological studies and considerable gaps in regional data on livestock in Portugal, which is an endemic area for *L. infantum* infection, the aim of the present study was to determine the presence of antibodies against *L. infantum* in the serum of sheep (*Ovis aries*) and goats (*Capra hircus*) in Portugal.

## Methods

### Study area

The study area comprised three main geographical regions of mainland Portugal: Trás-os-Montes (northwest), Centre and South (Table 1). Samples were collected from 19 municipalities: 12 in Trás-os-Montes, two in the Centre region and five in the South. A total of 87 different localities were identified within the three geographical regions.

**Table 1:** Seropositivity to *Leishmania infantum* in small ruminants from Portugal based on 22 categories of variables

Variable/categories	Animals tested,* n* (%)	Leishmania- positive animals, % (*n*)	95% Confidence interval
*Species*	2124 (100)	21.3 (453)	19.6–23.1
Sheep	1820 (85.7)	22.4 (407)	20.5–24.4
Goat	304 (14.3)	15.1 (46)	11.3–19.7
		*P* = 0.006*^a^	
*Adult age*	2124 (100)	21.3 (453)	19.6–23.1
No	218 (10.3)	18.8 (41)	13.9–24.6
Yes	1906 (89.7)	21.6 (412)	19.8–23.5
		*P* = 0.383^b^	
*Sex*	2124 (100)	21.3 (453)	19.6–23.1
Female	2003 (94.3)	21.4 (429)	19.6–23.3
Male	121 (5.7)	18.8 (24)	13.1–28.1
		*P* = 0.765^c^	
*Autochthonous breed*	2124 (100)	21.3 (453)	19.6–23.1
No	1135 (53.4)	24.9 (283)	22.4–27.6
Yes	989 (46.6)	17.2 (170)	14.9–19.7
		*P* < 0.001*^d^	
*Region*	2124 (100)	21.3 (453)	19.6–23.1
Trás-os-Montes	1832 (86.3)	21.8 (400)	20.0–23.8
Centre	37 (1.7)	10.8 (4)	3.0–25.4
South	255 (12.0)	19.2 (49)	14.6–24.0
		*P* = 0.183^e^	
*Herd size (n animals)*	2124 (100)	21.3 (453)	19.6–23.1
< 100	778 (36.6)	19.7 (153)	16.9–22.6
≥ 100	1346 (63.4)	22.3 (300)	21.1–24.6
		*P* = 0.172^f^	
*Herd type*	2124 (100)	21.3 (453)	19.6–23.1
Meat	1672 (78.7)	21.5 (359)	19.5–23.5
Milk	452 (21.3)	20.8 (94)	17.2–24.8
		*P* = 0.806^g^	
*Production system*	2124 (100)	21.3 (453)	19.6–23.1
Extensive	181 (8.5)	23.2 (42)	17.3–30.0
Semi-extensive	1887 (88.8)	21.5 (405)	19.6–23.4
Intensive	56 (2.6)	10.7 (6)	4.0–21.9
		*P* = 0.125^h^	
*Contact between goats and sheep*	2124 (100)	21.3 (453)	19.6–23.1
No	1282 (60.4)	23.2 (297)	20.9–25.6
Yes	842 (39.6)	18.5 (156)	16.0–21.3
		*P* = 0.012*^i^	
*Contact with other domestic species*	1851 (87.1)	21.8 (404)	20.0–23.8
No	1050 (49.4)	16.3 (171)	14.1–18.7
Yes	801 (37.7)	29.1 (233)	26.0–32.4
		*P* < 0.001*^j^	
*Barn type*	1851 (87.1)	21.8 (404)	20.0–23.8
Precarious	787 (37.1)	25.4^k^ (200)	22.4–28.6
Shelter	385 (18.1)	20.0 (77)	16.1–34.4
Modern	679 (32.0)	18.7^k^ (127)	15.8–21.8
		*P* = 0.005*^l^	
*Confinement system*	1851 (87.1)	21.8 (404)	20.0–23.8
Permanent confinement	55 (2.6)	18.2 (10)	9.1–30.9
Night confinement all year round	1188 (55.9)	24.6^m^ (292)	22.2–27.1
Night confinement only in winter	608 (28.6)	16.8^m^ (102)	13.9–20.0
		*P* < 0.001*^n^	
*Ventilation*	2124 (100)	21.3 (453)	19.6–23.1
Low aeration	964 (45.4)	19.0 (183)	16.6–21.6
High aeration	1160 (54.6)	23.3 (270)	20.9–25.8
		*P* = 0.019*^o^	
*Farm hygiene*	1851 (87.1)	21.8 (404)	20.0–23.8
Good	702 (33.1)	17.7^p,q^ (124)	14.9–20.7
Average	879 (41.4)	23.7^q^ (208)	20.9–26.6
Bad	270 (12.7)	26.7^p^ (72)	21.5–32.4
		*P* = 0.002*^r^	
*Drinking fountain hygiene*	1851 (87.1)	21.8 (404)	20.0–23.8
Good	696 (32.8)	16.7^s^^,t^ (116)	14.0–19.7
Average	859 (40.4)	21.8^s^^,u^ (187)	19.1—24.7
Bad	296 (13.9)	34.1^t,u^ (101)	28.7–39.8
		*P* < 0.001*^v^	
*Sharing of drinking fountains*	1851 (87.1)	21.8 (404)	20.0–23.8
No	964 (45.4)	24.5 (236)	21.8–27.3
Yes	887 (41.8)	18.9 (168)	16.4–21.7
		*P* = 0.005*^w^	
*Disinfection frequency*	1851 (87.1)	21.8 (404)	20.0–23.8
Never	343 (16.1)	19.5 (67)	15.5–24.1
Quarterly	496 (23.4)	24.0^x^ (119)	20.3–28.0
Biannual	661 (31.1)	25.9^y^ (171)	22.6–29.4
Annual	351 (16.5)	13.4^x,y^ (47)	10.0–17.4
		*P* < 0.001*^z^	
*Maternity area*	1851 (87.1)	21.8 (404)	20.0–23.8
No	1253 (59.0)	17.5 (219)	15.4–19.7
Yes	598 (28.2)	30.9 (185)	27.3–34.8
		*P* < 0.001*^aa^	
*Use of equipment from other farms*	2124 (100)	21.3 (453)	19.6–23.1
No	1315 (61.9)	25.1 (330)	22.8–27.5
Yes	809 (38.1)	15.2 (123)	12.8–17.9
		*P* < 0.001*^bb^	
*Veterinary assistance*	2124 (100)	21.3 (453)	19.6–23.1
Pre-defined prophylactic plan	141 (6.6)	14.9 (21)	9.5–21.9
Vaccination and deworming	1466 (69.0)	23.0 (337)	20.9–25.2
Brucellosis screening only	517 (24.3)	18.4 (95)	15.1–22.0
		*P* = 0.014*^cc^	
*Participation in competitions and/or animal markets*	2124 (100)	21.3 (453)	19.6–23.1
No	1915 (90.2)	21.9 (419)	20.1–23.8
Yes	209 (9.8)	16.3 (34)	11.5–22.0
		*P* = 0.073^dd^	
*Year*	2124 (100)	21.3 (453)	19.6–23.1
2020	505 (23.8)	14.7^ee^ (74)	11.7–18.0
2021	836 (39.4)	19.9^ff^ (166)	17.2–22.7
2022	510 (24.0)	32.2^ee,ff,gg^ (164)	28.1–36.4
2023	273 (12.9)	17.9^gg^ (49)	13.6–23.0
		*P* < 0.001*^hh^	
*Season*	2124 (100)	21.3 (453)	19.6–23.1
May–October	779 (36.7)	19.1 (149)	16.4–22.1
November–April	1345 (63.3)	22.6 (304)	20.4–24.9
		*P* = 0.067^ii^	
Total	2124 (100)	21.3 (453)	19.6–23.1

Pairwise comparisons incorporated Bonferroni correction (i.e. multiplying each *P* value by the number of potential comparisons). Only statistically significant differences are shown for second-level pairwise comparisons. The period May–October is considered to be the phlebotomine sand fly season, and the period November–April is considered to be the sand fly-free season

*df* Degrees of freedom, *χ*^2^ Chi-square test

*Statistically significant (*P* < 0.05)

^a^*χ*^2^ = 7.69, *df* = 1

^b^*χ*^2^ = 0.76, *df* = 1

^c^*χ*^2^ = 0.09, *df* = 1

^d^*χ*^2^ = 20.30, *df* = 1

^e^*χ*^2^ = 3.40, *df* = 2

^f^*χ*^2^ = 1.87, *df* = 1

^g^*χ*^2^ = 0.06, *df* = 1

^h^*χ*^2^ = 4.16, *df* = 2

^i^*χ*^2^ = 6.25, *df* = 1

^j^*χ*^2^ = 42.90, *df* = 1

^k^*χ*^2^ = 9.08, *df* = 1, *P* = 0.009*

^l^*χ*^2^ = 10.57, *df* = 2

^m^*χ*^2^ = 17.11, *df* = 1, *P* < 0.003*

^n^*χ*^2^ = 14.79, *df* = 2

^o^*χ*^2^ = 5.53, *df* = 1

^p^*χ*^2^ = 9.26, *df* = 1, *P* = 0.006*

^q^*χ*^2^ = 8.11, *df* = 1, *P* = 0.012*

^r^*χ*^2^ = 12.56, *df* = 2

^s^*χ*^2^ = 6.06, *df* = 1, *P* = 0.042*

^t^*χ*^2^ = 36.01, *df* = 1, *P* < 0.003*

^u^*χ*^2^ = 17.29, *df* = 1, *P* < 0.003*

^v^*χ*^2^ = 37.09, *df* = 2

^w^*χ*^2^ = 7.99, *df* = 1

^x^*χ*^2^ = 14.00, *df* = 1, *P* < 0.006*

^y^*χ*^2^ = 20.39, *df* = 1, *P* < 0.006*

^z^*χ*^2^ = 23.39, *df* = 3

^aa^*χ*^2^ = 42.19, *df* = 1

^bb^*χ*^2^ = 28.62, *df* = 1

^cc^*χ*^2^ = 8.57, *df* = 2 (no statistically significant differences in pairwise comparisons after the Bonferroni’s correction)

^dd^*χ*^2^ = 3.21, *df* = 1

^ee^*χ*^2^ = 42.34, *df* = 1, *P* < 0.006*

^ff^*χ*^2^ = 25.24, *df* = 1, *P* < 0.006*

^gg^*χ*^2^ = 17.42, *df* = 1, *P* < 0.006*

^hh^*χ*^2^ = 51.99, *df* = 3

^ii^*χ*^2^ = 3.24, *df* = 1

### Data and blood sample collection

To determine the presence of anti-*Leishmania infantum* antibodies in small ruminant flocks, serum samples were collected in serum tubes. Blood samples were obtained by puncturing the jugular vein using a vacutainer system with BD Vacutainer® PrecisionGlide™ 18 G × 1″ (1.2 × 25 mm) needles and VACUTEST® 4-ml serum tubes (BD, Franklin Lakes, NJ, USA). The samples were subsequently divided into duplicate samples and stored individually at − 20 °C in 1.5-ml tubes.

Data were obtained from farms in the sampling area on small ruminant species, including possible adult age, sex, possible autochthonous breed, geographical region, herd size and type, production system, contact between goats and sheep, contact with other domestic species, barn type, confinement system, ventilation/aeration, farm hygiene, drinking fountain hygiene, possible sharing of drinking fountains, disinfection frequency, existence of maternity wards, use of equipment from other farms, veterinary assistance, participation in competitions and/or animal markets and year and season of sample collection (Table 1).

Airflow conditions were classified into two levels: (i) low, where restricted openings and poor natural circulation resulted in insufficient aeration; and (ii) high: where barns featured optimised openings and/or automated mechanical systems providing continuous air exchange. To assess farm hygiene, we employed a three-tier qualitative scale: (i) good, characterised by optimal sanitary conditions with routine disinfection and minimal organic load; (ii) average, with intermediate cleanliness with intermittent manure removal; and (iii) bad, characterised by substandard hygiene marked by persistent waste accumulation and infrequent cleaning interventions. Similarly, drinking fountain hygiene was classified as “good”, “average” or “bad” based on the cleanliness and maintenance of the water sources: (i) good, characterised by clean drinking fountains with regular renewal and absence of visible impurities; (ii) Average, with moderate cleanliness with occasional presence of physical debris; and (iii) bad, characterised by poor sanitary status featuring stagnant water and evident accumulation of organic matter or biofilms. Hygiene categories were based on the DGAV (Portuguese National Authority for Animal Health) guidelines and EU Regulation (EC) No 852/2004. “Good” hygiene reflects full compliance with these legal sanitary standards, while “Bad” hygiene indicates a failure to meet minimum requirements for waste management and facility maintenance.

### Detection of antibodies to *L. infantum* by in-house enzyme-linked immunosorbent assay

An enzyme-linked immunosorbent assay (ELISA) for detecting antibodies against *L. infantum* was performed on all serum samples using the protocol described previously [17]. For the in-house ELISA (sensitivity of 99.37% and specificity of 97.50%), the crude antigen (strain MHOM/FR/78/LEM75 belonging to *L. infantum* zymodeme MON-1) was adjusted to a concentration of 20 µg/ml with phosphate-buffered saline (PBS). Briefly, each plate was coated with 100 µl/well of the 20 µg/ml antigen solution in 0.1 M carbonate/bicarbonate buffer and incubated overnight at 4 °C. A 100-µl aliquot of sheep sera, diluted 1:100 in PBS containing 0.05% Tween 20 (PBST) and 1% dry skimmed milk (PBST-M) as a blocking agent, was added to each well. The plates were incubated for 1 h at 37 °C in a moist chamber. After washing the plates for 3 min 3 times with PBST followed by one wash with PBS for 1 min, 100 µl of Protein A/G conjugated to horseradish peroxidase (Thermo Fisher Scientific, Waltham, MA, USA) diluted 1:10,000 in PBST-M was added per well. The standardisation of the ideal concentration of serum dilution and conjugated dilution was based on a previous case report of leishmaniosis in a small ruminant. The plates were incubated for 1 h at 37 °C in the moist chamber and then washed again with PBST and PBS as described above. The substrate solution (ortho-phenylene-diamine) and stable substrate buffer (Thermo Fisher Scientific) was added at 100 µl per well and developed for 20 ± 5 min at room temperature in the dark. The reaction was stopped by adding 100 µl of 2.5 M H_2_SO_4_ to each well. Absorbance values were read at 492 nm in an automatic microELISA reader (Microplate Photometer Biosan Hipo MPP-96; SIA BioSan, Riga, Latvia). Included on each plate were positive control samples from a clinically diagnosed seropositive dog, cat and goat with leishmaniosis [5], and samples from apparently healthy seropositive sheep. The optical density (OD) values for these positive controls exceeded 1.1 OD units, while negative controls, comprising serum from healthy, non-infected sheep and non-infected goat, exhibited OD values < 0.10 units. The same positive and negative sera were used for all assays, and the plates with an inter-assay variation > 10% were discarded All samples were tested in duplicate. The cut-off was set to 0.38 OD units (mean + 3 standard deviations [SD] of values from 90 sheep from non-endemic areas such as northern Spain), and results exceeding this value were considered positive. These 90 sheep were classified as healthy according to LeishVet guidelines [22]. This classification was based on a complete physical examination and the absence of clinicopathological abnormalities detected by routine red blood cell count (Idexx Procyte Dx; IDEXX Laboratories, Inc. , Westbrook, ME, USA), clinical chemistry (AmiShield; Protect Life International Biomedical Inc., Taoyuan City, Taiwan), urinalysis and serum protein electrophoresis by the agarose gel electrophoresis system (Hydragel Kit 1–2; Sebia, Evry, France). Laboratory parameters of these animals were not considered to be altered because they were within the reference intervals. Additionally, to evaluate diagnostic specificity, we included a panel of sheep serum samples infected with other pathogens (*Anaplasma ovis*, Bluetongue virus, *Brucella* spp., Foot-and-mouth disease virus, Epizootic hemorrhagic disease virus, Peste des petits ruminants virus and Rift Valley fever virus); all samples in this group tested negative by ELISA.

### Statistical analysis

The data obtained from the entire population were analysed using descriptive statistical methods. Exact binomial 95% confidence intervals (CI) were calculated for seroprevalence values. The Chi-square test (*χ*^2^) was used to compare the proportions of positivity among categories. Independent or explanatory variables with a significant difference between categories (probability [*P*] value < 0.05) were selected for multivariable logistic regression analysis to identify independent risk factors for seropositivity, by calculating odds ratios (OR) and their 95% CI [23]. Data from 2023, corresponding to 273 small ruminants, were not included in the logistic regression due to a lack of information on most of the 15 selected variables (Table 2), which meant that only a subset of 1851 animals could be analysed in the multivariable logistic regression. Median and interquartile range (IQR) values were calculated for the ELISA OD values. The Kolmogorov–Smirnov test was used to test for normality; and the Mann–Whitney U-test (MWU) was used to compare medians. Statistical significance was set at *P* < 0.05. Statistical analyses were conducted using IBMS SPSS Statistics 30.0® software (IBM Corp., Armonk, NY, USA).

**Table 2:** Identification of risk factors for seropositivity to *Leishmania infantum* in 1851 small ruminants from Portugal (1620 sheep and 231 goats) by multiple logistic regression

Variable/categories	Seroprevalence (%)	Odds ratio	95% Confidence interval
*Species*			
Goat	17.7	1 (Reference)	
Sheep	22.4	1.220 (*P* = 0.376)	0.785–1.897
*Autochthonous breed*			
Yes	17.6	1 (Reference)	
No	25.4	2.163 (*P* < 0.001*)	1.624–2.881
*Contact between goats and sheep*			
Yes	19.7	1 (Reference)	
No	23.1	1.920 (*P* < 0.001*)	1.360–2.713
*Contact with other domestic species*			
No	16.3	1 (Reference)	
Yes	29.1	1.435 (*P* = 0.057)	0.989–2.081
*Barn type*			
Modern	18.7	1 (Reference)	
Shelter	20.0	0.903 (*P* = 0.671)	0.565–1.445
Precarious	25.4	0.901 (*P* = 0.562)	0.634–1.281
*Confinement system*			
Night confinement only in winter	16.8	1 (Reference)	
Permanent confinement	18.2	0.415 (*P* = 0.045*)	0.176–0.979
Night confinement all year round	24.6	1.255 (*P* = 0.242)	0.858–1.835
*Ventilation*			
Low aeration	18.1	1 (Reference)	
High aeration	25.6	1.964 (*P* < 0.001*)	1.411–2.733
*Farm hygiene*			
Good	17.7	1 (Reference)	
Average	23.7	1.785 (*P* = 0.103)	0.890–3.583
Bad	26.7	1.171 (*P* = 0.505)	0.736–1.862
*Drinking fountain hygiene*			
Good	16.7	1 (Reference)	
Average	21.8	6.221 (*P* < 0.001*)	3.114–12.426
Bad	34.1	1.975 (*P* = 0.003*)	1.254–3.109
*Sharing of drinking fountains*			
Yes	18.9	1 (Reference)	
No	24.5	1.366 (*P* = 0.062)	0.985–1.895
*Disinfection frequency*			
Annual	13.4	1 (Reference)	
Never	19.5	1.215 (*P* = 0.472)	0.714–2.066
Quarterly	24.0	1.602 (*P* = 0.061)	0.978–2.626
Biannual	25.9	2.459 (*P* < 0.001*)	1.556–3.887
*Maternity area*			
No	17.5	1 (Reference)	
Yes	30.9	1.420 (*P* = 0.062)	0.982–2.053
*Use of equipment from other farms*			
Yes	14.9	1 (Reference)	
No	25.6	2.189 (*P* < 0.001*)	1.588–3.017
*Veterinary assistance*			
Pre-defined prophylactic plan	15.6	1 (Reference)	
Brucellosis screening only	19.0	0.798 (*P* = 0.519)	0.401–1.586
Vaccination and deworming	23.6	0.798 (*P* = 0.485)	0.423–1.505
*Year*			
2020	14.7	1 (Reference)	
2021	19.9	1.303 (*P* = 0.266)	0.818–2.078
2022	32.2	1.153 (*P* = 0.594)	0.683–1.948

*Statistically significant (*P* < 0.05)

## Results

### Animals studied

The number of studied sheep was 1820 and the number of studied goats was 304, totalling 2124 animals (Table 1). The number of sheep per municipality ranged from zero to 346, and that of goats from zero to 57. In addition, the number of sheep per locality ranged from zero to 49, and that of goats from zero to 37. The descriptions of variables and categories, their corresponding positivity/seroprevalence values, and the results of statistical analyses are presented in Tables 1 and 2.

### Seroprevalence of . *infantum* and associated factors

Overall seroprevalence was 21.3% (453/2,124; 95% CI 19.6–23.1), with higher seropositivity in sheep than goats (22.4% vs 15.1%). In the analysis of 2124 animals, there was a statistically significant difference for both species and contact between goats and sheep (*χ*^2^ = 7.69, *df* = 1, *P* = 0.006 and *χ*^2^ = 6.25, *df* = 1, *P* = 0.012, respectively; Table 1). However, in the subset of 1851 animals used for the multivariable logistic regression analysis, these differences were no longer significant (species: *χ*^2^ = 2.31, *df* = 1, *P* = 0.129; contact: *χ*^2^ = 2.69, *df* = 1, *P* = 0.101). Despite this, species and contact between goats and sheep were included in the multivariable logistic regression analysis (Table 2) because they were significant in the full analysis of 2124 animals. No other variable showed this pattern.

The median of ELISA OD values for the 2124 animals was 0.24 (IQR 0.15–0.36). The positive animals had a median OD of 0.50 (IQR 0.43–0.62) and the negative ones a median OD of 0.20 (IQR 0.14–0.27). A statistically significant difference was found between these two medians (MWU, *U* = 756600.50, *Z* = 32.66, *P* < 0.001). In addition, sheep had a median OD of 0.24 (IQR 0.16–0.36) and goats a median OD of 0.22 (IQR 0.15–0.31), with the difference also being statistically significant (MWU, *U* = 305730.50, *Z* = 2.94, *P* = 0.003). Animals sampled from May to October (regarded as the phlebotomine sand fly season) had a median OD of 0.22 (IQR 0.14–0.34), while those sampled from November to April (sand fly-free season) had a median OD of 0.25 (IQR 0.16–0.36; MWU, *U* = 470746.0, *Z* = − 3.90, *P* < 0.001).

## Discussion

In the present study we analysed the seroprevalence of leishmaniosis in small ruminants in Portugal, recording a global seroprevalence of 21.3%. The dog is the leading domestic reservoir of *L. infantum* infection in Europe. In Portugal, serological surveys in dogs have reported an overall seroprevalence ranging from 6.5% in 2012 [24] to 12.5% with marked geographical heterogenicity and substantially higher values in specific areas in 2021 [20]. The latter report disclosed a canine seroprevalence of 15.7% for the Bragança district [20], which mainly coincides geographically with the Trás-os-Montes region sampled in the present study. The canine seroprevalence reported by Almeida et al. [20] was 12.5% for the Centre region and 16.1% for the Alentejo region, which geographically encompass the Centre and South regions of the present study, respectively. The seroprevalence values in dogs are lower than those we found in small ruminants, i.e. 21.8% (Trás-os-Montes), 13.8% (Centre) and 19.2% (South), but the serological techniques and their cut-offs were different in both studies (present study and [20]). In the present study an ELISA was run on serum, and Almeida et al. used the direct agglutination test (DAT) on blood on filter paper [20]. Nevertheless, numerous animal species, both domestic and wild, could contribute to the maintenance of the parasite in nature and to the occurrence of human leishmaniasis [11]. The number of potential reservoirs is increasing and among these new reservoirs are large animals in which the presence of *L. infantum* has been demonstrated, such as horses [25] and donkeys [26].

Although limited information is currently available on the potential role of small ruminants, such as sheep, in the *Leishmania* spp. transmission cycle, the parasite has been detected in small ruminants animals in non-European countries such as China [27], Ethiopia [28] and Iran [29]. The first European study designed to identify the seroprevalence of *L. infantum* in a sheep population in Europe was conducted in Greece, a region endemic for leishmaniosis [30]. The results of this study indicated a seroprevalence of 0.0%, as no positive animals were detected. A more recent seroprevalence study conducted in Spain, also an endemic area for leishmaniosis, reported a seroprevalence of 9.3% [17]. In contrast, in the federal states of Bavaria and Baden-Württemberg in southern Germany, where leishmaniosis is not an endemic disease, 1.5% of sheep were found to have antibodies against *L. infantum* [19]. However, the results of the most recent studies are not directly comparable with those of the study conducted in Greece.

In the multivariable model, the sheep–goat difference did not persist, indicating that increased seropositivity was better explained by management and environmental correlates rather than by species per se. Factors independently associated with increased odds of seropositivity included non-autochthonous breeds (OR 2.163), holdings without goat–sheep contact (OR 1.920), high aeration/ventilation (OR 1.964), poorer drinking-fountain hygiene (OR up to 6.221), biannual (vs annual) disinfection (OR: 2.459) and not using equipment from other farms (OR 2.189). In contrast, permanent confinement decreased the odds of seropositivity (OR 0.415).

Overall, increased seropositivity in small ruminants is primarily linked to farm management, hygiene and housing features that may influence sand fly exposure, rather than intrinsic animal factors. Because *L. infantum* transmission is vector-borne, the independent associations observed were mainly with farm-level variables that plausibly modify animal–sand fly contact (housing openness/ventilation, hygiene and a favourable microenvironment around farms and management typologies), whereas animal-level factors such as species did not remain significant after these confounders were controlled. Some of the identified risk factors (i.e. biannual barn disinfection vs annual disinfection; non-use of equipment from other farms; and absence of contact between goats and sheep) warrant careful interpretation. These associations may reflect the complex and context-dependent epidemiology of vector-borne diseases, in which commonly assessed biosecurity-related variables do not necessarily capture all of the relevant ecological and entomological determinants of transmission.

Although the variables included are epidemiologically relevant and widely applied in livestock health research, the scope of the present study does not allow for a detailed assessment of all mechanisms potentially influencing vector exposure and pathogen circulation. In particular, factors related to animal contact patterns and equipment sharing may have a different or indirect role in the epidemiology of vector-borne infections [31, 32]. Accordingly, the observed associations should be regarded as exploratory and as contributing to hypothesis generation, supporting the need for further studies integrating environmental, entomological, and management-related data to refine the interpretation of these findings.

In our study, the seroprevalence of leishmaniosis in small ruminants varied by species (sheep or goats). The presence of anti-*Leishmania* antibodies was higher (22.4%) in sheep than in goats (15.1%), and the tested sheep also had a higher median of ELISA OD values than the goats. The animals sampled outside of the sand fly season had higher OD values, which may indicate more prolonged immune stimulation, potentially due to more frequent contact with infected vectors and possible established infections. Moreover, statistical differences in seropositivity were detected between autochthonous and non-autochthonous breeds, with higher seropositivity in non-autochthonous breeds. Although there is no information available on small ruminants, in the case of *L. infantum* infection in dogs, the activation of T helper type 1 cells, which are associated with resistance to infection, or T helper type 2 cells, which are associated with disease progression, may differ depending on the canine breed [33], especially in autochthonous breeds, which suggests a natural resistance in the Ibizan Hound [34] or Cirneco dell’Etna [35] against *L. infantum* when compared with other susceptible dog breeds.

For this reason, the potential use of sheep as sentinels for *L. infantum* infection in certain areas is worth considering [19]. A sentinel animal is a species used to detect risks and, in this context, to provide early warning of potential threats. Given that sheep develop antibodies upon exposure to *L. infantum,* flocks in certain regions, such as rural areas, could be used to identify new areas at risk of leishmaniosis early. Climate change is expected to have a positive impact on the cycle of leishmaniosis vectors, including in Portugal, leading to increased sand fly infections, particularly in the northern regions [36]. Rising temperatures enable the vector to establish itself in new habitats where it previously could not survive. Furthermore, the extension of the warm season extends the vectors’ activity period. The use of sheep as sentinels would not only help identify risk areas for leishmaniosis but also facilitate the indirect detection of new vector habitats, such as sand flies.

This epidemiological study demonstrates the exposure to *L. infantum* in Portugal, highlighting the importance of considering the role of production animals in the leishmaniosis cycle and their potential involvement in clinical leishmaniasis in other species, including humans. Further research is needed to determine whether sheep develop the disease and to assess the impact of sheep on animal health and public health in endemic regions.

## Conclusions

This cross-sectional serosurvey demonstrates widespread exposure of small ruminants to *L. infantum* in mainland Portugal, with an overall seroprevalence of 21.3% across three geographical regions. Although seropositivity differed between sheep and goats, species was not independently associated with seropositivity after adjustment, indicating that exposure is better explained by farm-level and management-related determinants. In the multivariable analysis, non-autochthonous breeds and several husbandry and biosecurity variables were associated with increased odds of seropositivity, whereas permanent confinement was protective, supporting the relevance of practices that may modulate contact with phlebotomine sand flies. These findings highlight that production animals in endemic settings are frequently exposed to *L. infantum* and that risk is closely linked to modifiable management conditions. Further longitudinal and parasitological studies are warranted to clarify whether seropositive sheep and goats can develop clinical disease, to evaluate the frequency and duration of parasitemia and infectiousness to vectors and to define the implications of small ruminant exposure for integrated animal and public health surveillance in Portugal.

## Data Availability

Data supporting the main conclusions of this study are included in the manuscript.

## Abbreviations

ELISA: Enzyme-linked immunosorbent assay
MWU: Mann–Whitney U-test
OD: Optical density
